# High-Grade Serous Ovarian Cancer: Basic Sciences, Clinical and Therapeutic Standpoints

**DOI:** 10.3390/ijms20040952

**Published:** 2019-02-22

**Authors:** Michael-Antony Lisio, Lili Fu, Alicia Goyeneche, Zu-hua Gao, Carlos Telleria

**Affiliations:** Experimental Pathology Unit, Department of Pathology, McGill University, Montreal, QC H3A 2B4, Canada; michael-antony.lisio@mail.mcgill.ca (M.-A.L.); Lili.Fu@muhc.mcgill.ca (L.F.); alicia.goyeneche@mcgill.ca (A.G.); zu-hua.gao@mcgill.ca (Z.-h.G.)

**Keywords:** high-grade serous ovarian cancer, cortical inclusion cysts, serous tubular intra-epithelial carcinoma, ovarian surface epithelium, homologous recombination, BRCA, mutant p53, genetically-engineered mouse models, debulking surgery, chemotherapy

## Abstract

Among a litany of malignancies affecting the female reproductive tract, that of the ovary is the most frequently fatal. Moreover, while the steady pace of scientific discovery has fuelled recent ameliorations in the outcomes of many other cancers, the rates of mortality for ovarian cancer have been stagnant since around 1980. Yet despite the grim outlook, progress is being made towards better understanding the fundamental biology of this disease and how its biology in turn influences clinical behaviour. It has long been evident that ovarian cancer is not a unitary disease but rather a multiplicity of distinct malignancies that share a common anatomical site upon presentation. Of these, the high-grade serous subtype predominates in the clinical setting and is responsible for a disproportionate share of the fatalities from all forms of ovarian cancer. This review aims to provide a detailed overview of the clinical-pathological features of ovarian cancer with a particular focus on the high-grade serous subtype. Along with a description of the relevant clinical aspects of this disease, including novel trends in treatment strategies, this text will inform the reader of recent updates to the scientific literature regarding the origin, aetiology and molecular-genetic basis of high-grade serous ovarian cancer (HGSOC).

## 1. Prevalence and Mortality

Ovarian cancer is a salient public health concern, which, in spite of its infrequent incidence, remains the deadliest form of gynaecological malignancy. According to the WHO, each year an estimated total of 225,500 cases of ovarian cancer will be diagnosed and 140,200 patients will succumb to this disease, representing the 7th most common form of cancer and the 8th leading cause of cancer-related death among women worldwide [1,2]. These figures, taken together, underline the status of ovarian cancer as significant source of morbidity and mortality in the global population. In Western nations, ovarian cancer is the 5th most frequent cause of cancer-related death in women [3]. The Surveillance, Epidemiology and End Results (SEER) program of the American National Cancer Institute (NCI) records an annual incidence of 11.6 cases/100,000 women per year according to the latest statistical cohort, with an estimated 224,940 women living with the disease in 2015 [4]. In Canada, the Canadian Cancer Society predicted in 2017 an average of 2800 cases diagnosed and 1800 deaths/year [5]. Whereas the survival rates for a number of solid tumours have improved significantly in the last 50 years, a recent meta-analysis drawing upon survival data from numerous countries concluded that the 5-year overall survival from ovarian cancer had remained virtually unchanged since about 1980 [6]. According to the most recent figures published by the SEER (2008–2014), the current 5-year survival rate in the US is approximately 47.4% [4].

## 2. Subtype Classification

Although the term “ovarian cancer” implies a unitary disease, from the perspective of the pathologist it was apparent as early as the 1930s that it was more appropriate to classify ovarian neoplasms as multiple distinct entities through the lens of histopathology [7]. This culminated in the 1973 WHO guidelines, which signified the first systematic attempt to delineate the many ovarian cancer subtypes [7]. Histologically, about 90% of ovarian tumours are deemed to have occurred through the transformation of epithelial cells as opposed to those originating from germ cells or sex-cord-stromal tissues [8]. These are thus designated as epithelial ovarian cancers (EOC). That nomenclature itself applies to a broad category of disease with a whole range of taxonomy therein contained. This notably includes the four well-defined histological subtypes, which have constituted the basis for EOC diagnosis over the past few decades. These are referred to as: serous, mucinous, clear-cell and endometrioid—appellations deriving from their morphology and tissue architecture as observed through microscopy. Furthermore, the assignment of a tumour grade, based on the apparent degree of cytological aberration, allows for an additional degree of stratification for serous and endometrioid EOCs [3]. Thus, despite sharing some similarity in histological appearance and terminology, high-grade and low-grade serous carcinomas of the ovary are now considered to be two entirely different neoplasms, with distinct modes of carcinogenesis, molecular-genetic features and sites of origin [9]. While the majority of cases observed clinically belong to one of the four major histotypes, a number of rarer types have been noted. These include malignant transitional cell (Brenner) tumours as well as cases of mixed type and undifferentiated carcinoma [10].

Although referred to as ovarian cancer, it has long been observed that the histology of these tumours resembles non-ovarian tissues. For example, endometrioid ovarian carcinoma, as its name suggests, features a glandular architecture similar to the endometrium, while mucinous tumours can resemble either endocervical glands or the gastrointestinal epithelium [11]. Recent studies have supported the notion of an extra-ovarian origin for many mucinous tumours along with the carcinomas of the clear-cell and endometrioid subtypes, which likely derive from metastatic intestinal tumours and endometriotic lesions respectively [12,13]. The origin of the serous subtype was long debated but in the case of high-grade serous neoplasms, it is now widely acknowledged that the majority originate from the epithelium of the fallopian tube.

Recent efforts to study EOC from the molecular and genetic perspective have led to a paradigm shift in the classification of this disease via the introduction of the dualistic model of ovarian carcinogenesis. This model was first proposed by Kurman and Shih in 2004 and has since garnered widespread acceptance, being officially recognized in 2014 by the WHO in their updated classification guidelines for tumours of the female reproductive organs [9,14]. This model segregates the variety of EOC subtypes into two broad categories called Type 1 and Type 2. The Type 1 neoplasms typically develop along a step-wise progression from pre-malignant or borderline lesions in a manner common to many other epithelial cancers [10]. From the genetic perspective, these tumours display frequent oncogenic alterations to many cellular signalling pathways such as RAS-MAPK and PI3K-AKT but are otherwise genomically stable and P53 wild type [10]. From a clinical perspective, these tumours typically present as large, unilateral, cystic neoplasms that grow in an indolent fashion and when confined to the ovary they have an excellent prognosis [10]. This category includes low-grade serous, clear-cell, mucinous and transitional cell (Brenner) subtypes [10].

By contrast, the Type 2 category is marked by a far more aggressive pattern of disease behaviour. Tumours develop rapidly and usually are disseminated widely at the time of presentation, resulting in poor overall prognosis [10]. From a genetic viewpoint, these tumours are characterized by P53 mutations and genomic instability due to defects in pathways contributing to DNA repair [10]. The prototypical Type II neoplasm, HGSOC, is by far the dominant subtype diagnosed clinically and accounts for 70–80% of deaths from all forms of ovarian cancer [10,15]. In summary, the term ovarian cancer is a broad designation for a myriad of distinct diseases sharing an anatomical site upon presentation.

## 3. Histopathology

**Figure 1:**
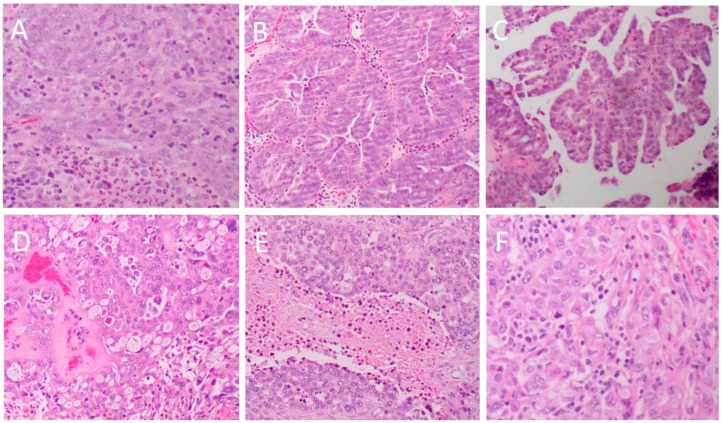
Heterogeneity of the histopathological architecture of high-grade serous cancer. (**A**): solid architecture (original magnification × 20); (**B**): glandular architecture with slit-like spaces (original magnification × 10; (**C**): papillary architecture (original magnification × 5); (**D**): cribriform and pseudoendometroid architecture (original magnification × 20); (**E**): solid architecture with “geographic” necrosis (original magnification × 10); (**F**): solid architecture with tumour infiltrating lymphocytes (original magnification × 20).

From the perspective of a pathologist visualizing stained tissue sections under a microscope, HGSOC tumours typically feature solid masses of cells (Figure 1A) with slit-like fenestrations (Figure 1B) [9]. In some areas, the tumours often have a papillary (Figure 1C), glandular (Figure 1B) or cribriform (Figure 1D) architecture that is said to resemble the surface epithelium of the fallopian tube [9,11]. The regions of solid growth are frequently accompanied by areas of extensive necrosis (Figure 1E) [9]. In certain cases, HGSOC may present with areas displaying a solid growth pattern that simulates the appearance of endometrioid or transitional cell carcinoma (Figure 1D) [10]. Although morphologically distinct, these tumours show an immunoreactivity identical to typical HGSOC and are thus not considered as a separate entity [10]. Researchers have recently named a group of HGSOC as the SET (“Solid, pseudo-Endometrioid and/or Transitional cell carcinoma-like”) tumours [16]. It was found that SET tumours frequently associate with BRCA1 mutations and contain a greater number of tumour-infiltrating lymphocytes (Figure 1F) compared to typical HGSOC [16].

**Figure 2:**
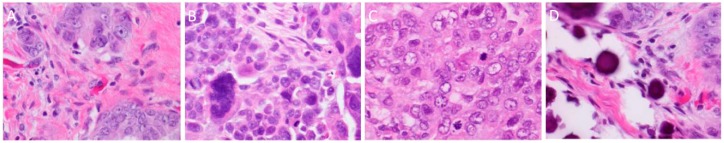
Cytological features of high-grade serous cancer. (**A**): Multinucleated tumor giant cells; (**B**): severe pleomorphism and prominent nucleoli; (**C**): frequent mitotic figures; (**D**): psammoma bodies. All original magnifications × 40.

From the cytological perspective, HGSOC is characterized by high-grade nuclear atypia; with large, hyperchromatic and pleomorphic nuclei with the potential for multinucleation (Figure 2A) [9]. The nucleoli are usually prominent and might appear large and eosinophilic (Figure 2B) [9]. There is usually a high mitotic index with an abundance of visible mitotic figures (Figure 2C) that may be of atypical appearance [9]. Psammoma bodies, which are areas of calcification typically associated with papillary tumours, are also typically present (Figure 2D) [9].

**Figure 3:**
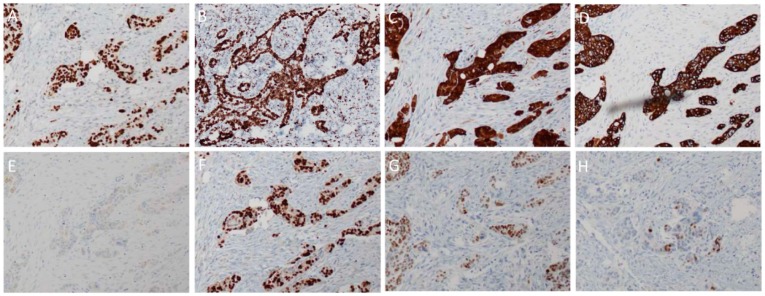
Immunological markers typically seen in high-grade serous ovarian cancer. (**A**): p53; (**B**): WT-1; (**C**): p16; (**D**): CK7; (**E**): CK20; (**F**): PAX8; (**G**): ER; (**H**): PR. All original magnifications × 10.

**Figure 4:**
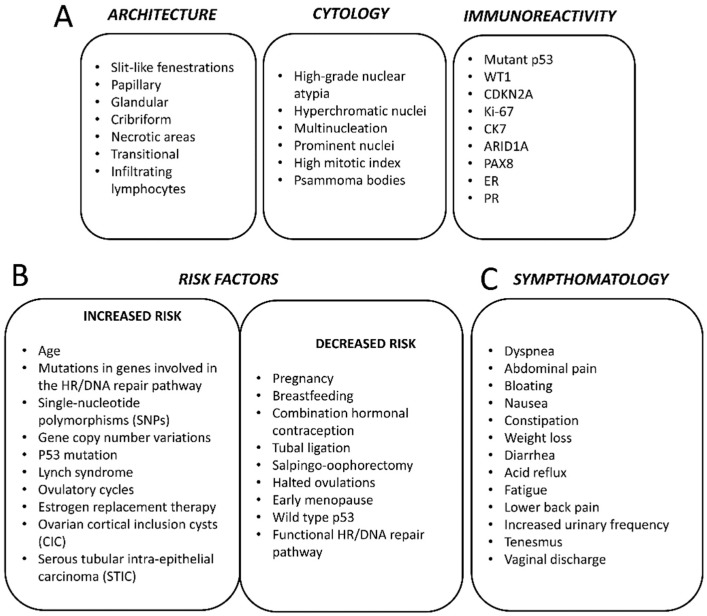
Histopathological features (**A**), risk factors (**B**) and symptoms (**C**) of high-serous ovarian cancer.

A number of immunological markers are used to differentiate HGSOC from other subtypes of EOC. Unlike low-grade serous tumours, HGSOC is almost invariably P53-mutant and will usually stain with a strong diffuse nuclear-positivity in nearly all cells (Figure 3A) [9]. This, however, is dependent on the type of mutation present. Missense mutations in TP53 typically correlate with positive staining due to the mutant protein accumulating owing to a lost capacity for degradation by the proteasome [9]. If, however, the gene contains a nonsense mutation, then the resultant truncated form of the protein might not be detectable by the antibody [9]. In that case, the staining would be almost totally negative. Compared to the alternative forms of EOC, HGSOC is frequently found to stain positive for WT1 (Figure 3B) and CDKN2A (a.k.a. P16) (Figure 3C) [9]. The proliferation index, assessed through the number of cells positive for Ki-67, would be higher compared to low-grade serous lesions (Figure 4A, right panel) [9]. The epithelial marker CK7 is positive in HGSOC (Figure 3D) but CK20 is usually negative (Figure 3E) [11]. Furthermore, when compared to ovarian clear-cell carcinoma, HGSOC is HNF1β negative and ARID1A positive (Figure 4A, right panel) [17]. In common with most other forms of EOC is the expression of PAX8 (Figure 3F), a marker of tissues of Müllerian origin including the fallopian tube [18]. The expression of the oestrogen receptor (ER) is detectable in about 80% of cases (Figure 3G), whereas the progesterone receptor (PR) is only found to be positive in around 30% of HGSOC patient samples (Figure 3H) [9].

## 4. Epidemiology and Risk Factors

The distribution of ovarian cancer incidence worldwide is not even, with a substantial variation on the basis of geography, ethnicity and the level of economic development. The peak age-adjusted incidence rates are in Northern and Central/Eastern Europe; with intermediate rates seen in North America, Western Europe and Australia; and lower rates in Asia and Africa [19]. Recent trends seem to show a stable or slight reduction in age-standardized rates in most high-income countries, whereas they appear to be rising in many low- and middle-income countries [19]. As such, there is far less of a disparity in incidence compared to 30 years ago [19]. In countries with a multi-ethnic population, the incidence may depend on ethnicity. For example, in the US the disease is more frequent among non-Hispanic, Caucasian women compared to Hispanic, Asian or African American women [4].

Like most other epithelial cancers, EOC tends to be diagnosed more frequently as a function of increasing age. As such, the number of cases occurring each year is expected to increase as global life-expectancies continue to improve [19]. In the US, the median age of diagnosis is at 63 years [4]. Epithelial ovarian cancer subtypes are infrequently seen in pre-menopausal women (≤45 years of age) while ovarian germ cell tumours occur mainly in younger women [19]. The total lifetime risk of developing ovarian cancer has been estimated to be only about 1.3% for American women; however, there are a number of known risk-factors that may modify the risk in individuals [4,19] (Figure 4B). For example, there is a substantial heritable component of risk due to genetic factors. The risk for women with an affected first-degree relative is threefold greater than that of women without any affected relatives [20]. Familial cases usually are found to be due to germline mutations in the tumour-suppressor genes BRCA1 and BRCA2, which also contribute increased risk of developing breast cancer in these same families [19]. A recent study found that 3.6% of ovarian cancer patients have germline mutations in BRCA1 while 3.3% have germline mutations in BRCA2 [21]. Overall, it was estimated that germline BRCA1 and BRCA2 mutations contribute to the development of 10–20% of EOCs [22]. Compared to the normal population, BRCA1 mutation carriers have an estimated 44% risk of developing ovarian cancer by age 70, while this risk is up to 27% for BRCA2 mutant individuals [23]. The cancers occurring in these women are usually high-grade serous carcinomas, which manifest at an earlier age than in sporadic cases [19]. The contribution of high-penetrance alleles of BRCA1/2 can only account for a small part of the heritable component of ovarian cancer [22]. Many other genes bearing low penetrance mutations are thought to play an important role. For instance, women with mutations in the genes BRIP1, RAD1C and RAD1D have estimated lifetime risks of developing ovarian cancer of 5.8%, 5.2% and 12%, respectively [24,25]. Other gene variations that have been linked with greater risk include BARD1, CHEK2, MRE11A, RAD50, PALB2 and ATM [25,26]. The common link between all these genes is their role in the homologous recombination (HR)-mediated pathway of DNA repair, which is known to play a prominent role in the pathophysiology of HGSOC. Women with Lynch syndrome bearing mutations in genes involved in DNA mismatch repair also have a greater risk of developing EOC, mostly of the clear-cell and endometrioid subtypes [27].

In recent years, genome-wide association studies (GWAS) have also been used to search for single-nucleotide polymorphisms (SNPs) correlating with greater risk of developing ovarian cancer [22]. Several of these loci have been identified, and, while each is associated with only a miniscule increase in absolute risk, the combination of multiple alleles has been demonstrated to considerably impact an individual’s polygenic risk score [28].

Endometriosis is known to predispose individuals towards developing EOC, particularly the clear-cell and endometrioid subtypes, which are known to derive from endometriotic lesions [13,19].

Many modifiable or lifestyle factors have also been viewed as influencing an individual’s risk of developing ovarian cancer. Generally, ovarian cancer has been associated with women having experienced a greater number of ovulatory cycles in their lifetime [19]. As such, factors tending to reduce a woman’s ability to ovulate have been linked with a reduced lifetime risk of developing this disease. For example, both the early occurrence of menarche and an older age at menopause have been connected with a possible increased risk [29,30]. Likewise, women who have given birth have a lower risk than nulliparous women, with a risk reduction of 10–20% associated with each additional birth [30]. Studies have also found that women who breastfeed have lower risk compared to those who do not [30,31]. The use of hormone (oestrogen plus a progestin)-containing oral contraceptives has been robustly associated with a reduced risk of developing ovarian cancer, with users or former users having up to 30% lower risk compared to never-users [19,32]. This apparent protective effect was more apparent in long-term users and extended to all major subtypes of EOC [30,32]. One study claimed that the use of these compounds might have even prevented up to 200,000 worldwide cases of EOC over the past several decades [32]. Procedures aimed at reducing fertility, such as by tubal ligation, have been shown to reduce the risk of developing certain forms of EOC, while the use of oestrogen hormone therapy during menopause has been linked with a heightened risk in the women undergoing such treatment [30,33,34] (Figure 4B).

Other potential risk factors include obesity, diabetes, smoking and usage of perineal talc [19]. Unfortunately, many of the modifiable factors that have been associated with ovarian cancer are not easily amenable to change, while others, including pregnancy and oral contraceptives use, cannot be recommended as a cancer prevention strategy [19]. Moreover, a recent study of an Australian cohort concluded that only about 7–11% of ovarian cancer cases could be attributed to these modifiable factors [35].

## 5. Origin

The precise cell and tissue of origin for HGSOC has long been a matter of contention: unlike low-grade serous tumours, which arise from pre-existing lesions such as serous cystadenomas or serous borderline tumours, locating a precancerous component in the case of HGSOC has proven difficult [36]. In fact, HGSOC remains the only epithelial cancer without a recognized precancerous lesion [11]. Because most HGSOC patients, even at an early stage, feature the cancerous involvement of the ovary, it was natural to assume that the disease originated from that location. The tissue of origin was reputed to be the ovarian surface epithelium (OSE), a simple, uncommitted layer of flat-to-cuboidal epithelium derived from the coelomic mesoderm and related to the mesothelial covering of the peritoneal cavity [11,37]. As early as 1971, Fathalla put forward the “incessant ovulation” hypothesis that would attain widespread acceptance in subsequent years [36,38]. His theory contended that the constant cycle of repair and regeneration the OSE experiences as a result of ovulation might contribute to carcinogenesis by creating the kind of pro-inflammatory and pro-oxidative microenvironment conducive to DNA damage [36,37,38]. The inability to repair such damage adequately was thought to be at the root of HGSOC carcinogenesis [36]. Indeed, it has since become known that patients harbouring germline mutations in BRCA1/2—which encode proteins implicated in the pathway responsible for the homologous recombination-mediated repair of double strand breaks (DSBs)—are at much greater risk of developing HGSOC [36]. According to this theory, the total number of ovulatory cycles a woman experiences would be related directly to her risk of acquiring HGSOC [36,38]: numerous studies concluded that factors suppressing ovulation, such as pregnancy, breastfeeding and the use of hormone-containing oral contraceptives, reduced an individual’s risk of developing the disease [36,37,39] (Figure 4B), thus reinforcing the theory.

Furthermore, it was observed that ovulatory repair resulted in the tendency for certain sections of the OSE to invaginate and become trapped beneath the surface of the ovary in structures called cortical inclusion cysts (CICs) [37]. Within the ovary, CICs are exposed to several hormones capable of promoting growth and differentiation, something thought sufficient to engender the transition to a state of metaplasia in the OSE lining of these CICs [37]. In cells harbouring pre-existent mutations or DNA damage, this would create the ideal scenario for neoplastic transformation [37].

The theory for an ovarian origin of HGSOC nevertheless remained questionable in a number of key areas. Histologically, HGSOC is said to resemble more closely tissues developmentally derived from the Müllerian duct during embryogenesis [11,39,40]. It was not known how the coelomic cells of the OSE could differentiate into a Müllerian-like tissue during carcinogenesis [11]. Some have postulated that the relatively undifferentiated nature of the OSE would allow it to more readily undergo metaplasia to resemble a Müllerian phenotype [39,40]. One study shows that the ectopic activation of the gene HOXA9 in cultured mouse OSE was sufficient to induce the formation of tumours histologically resembling serous carcinoma [41]. Although this study provided a potential mechanistic framework for an OSE origin of HGSOC, doubt nevertheless persisted due to the enduring absence of any identifiable precursor lesion or in-situ carcinoma in patients’ ovaries [11,39]; at the time, many speculated that it was due to their destruction during the process of tumorigenesis [42].

In 1999, Dubeau cast doubt on the dogma that HGSOC originated from the OSE and instead advocated for a source derived from the Müllerian epithelium [43]. His argument invoked the differences in histogenesis between the OSE and the Müllerian epithelium, of which the latter appears to resemble more closely the histological phenotype of ovarian carcinomas. Furthermore, he raised the lack of evidence supporting the theory of the OSE being capable of metaplastic transformation to resemble the Müllerian phenotype and emphasized on the continued absence of observable precursor lesions in the ovary. Moreover, he argued that cases of primary peritoneal carcinoma, which arise without ovarian involvement and which are indistinguishable clinically and histopathologically from HGSOC, were proof of an extra-ovarian cell of origin for this neoplasm [43]. He advanced the theory that serous ovarian carcinomas were derived from the secondary Müllerian system, the vestigial remnants of Müllerian epithelium present ectopically outside of the cervix, endometrium and fallopian tube [43]. Nonetheless, the belief that HGSOC could originate from a tissue of extra-ovarian origin was not widely held until the introduction of risk-reducing salpingo-oophorectomy in patients with inherited BRCA mutations [10]. In 2001, Piek et al. described the presence of small dysplastic lesions similar to HGSOC within the fallopian tubes of suspected BRCA mutation carriers [39,44]. Stratified, disorganized and enlarged epithelial cells with highly atypical nuclei morphologically characterized these lesions [39]. The examination of samples from a cohort of non-mutant individuals failed to locate any such lesions [36,44]. This discovery was aided by the introduction of a new histological approach for sampling the fallopian tube in which the entire fallopian tube, with particular attention to the fimbria, was sectioned [10]. Previously, studies examining the ovary for precursor lesions failed to completely examine the fallopian tube [7]. These lesions, which later became known as serous tubular intra-epithelial carcinomas (STICs), featured the virtual absence of ciliated cells with a shift in favour of the alternative secretory population [37]. Later studies have established that these lesions are far more common at the ciliated end of the fallopian tube, which is the section directly adjacent to the ovary [37,45]. An analysis using immunohistochemistry found that these lesions stained strongly for P53 compared to the surrounding epithelium, suggesting that these cells were P53 mutant [11,46]. They also over-expressed γH2AX, a marker of DNA double strand breaks [11,46]. One study found that 38% of a cohort of BRCA mutant women having undergone salpingo-oophorectomy harboured STIC lesions in their fallopian tubes, without any corresponding abnormalities being found in the ovaries of such patients [45]. The existence of these microscopic intra-epithelial carcinomas suggested that the secretory epithelial cells of the distal fallopian tube (FTSEC) were the preferred cells of origin for HGSOC, at least in women bearing BRCA1/2 mutations. This was supported strongly by a study by Kuhn et al. showing that STICs possessed the identical P53 mutation to that present within the concurrent HGSOC in women with this disease [47]. Furthermore, it has been shown that STICs contain shortened telomeres compared to the co-existent cancer within the same patient [48]. The presence of telomere shortening has become an established hallmark of the early stages of carcinogenesis [10].

While STICs were known to occur in women with BRCA mutations, it was not known if they could contribute to carcinogenesis in sporadic cases of HGSOC. A 2007 study by Kindelberger et al. showed that STICs were found in 52% of patients with sporadic advanced-stage HGSOC [46]. STICs also have been reported in the fallopian tubes of women undergoing hysterectomy and bilateral salpingo-oophorectomy for non-prophylactic reasons [10,49]. Recent genetic studies have established that HGSOC and paired STICs have many other shared genetic alterations including changes in gene copy number [22]. One study established that, in cases of HGSOC featuring the amplification of CCNE1, a similar copy number change for this gene was present in the STICs isolated from the same patient [50]. The tubal origin for HGSOC also has been reinforced by a study that used gene expression profiling to show that the pattern of gene expression in HGSOC more closely resembles that of the fallopian tube epithelium rather than that of the OSE [51]. Transformation of cultured human fallopian tube epithelium in-vitro also results in cells that resemble the morphology, immunophenotype and gene expression profile of human HGSOC [52]. In addition, a novel mouse model that features induced mutations in the same genes commonly affected in human patients also develops serous carcinoma from the fallopian tube [53].

Why the overwhelming majority of STICs are found to occur at the fimbriated end of the fallopian tube remains a mystery [42]. Some studies claim to show that the fimbriae are enriched in cells with “stem like” properties [54]. Wang and colleagues [55] used lineage tracing in a murine model to identify a population of quiescent, label-retaining cells in the distal oviduct of female animals. There were subsequently shown to be endowed with enhanced spheroid forming capacity along with the ability to differentiate into structures resembling multiples tissues of Müllerian origin, including the endometrium and distal/proximal oviduct. The authors theorize that their results point to the existence (in humans) of a stem-like cell of origin in the distal fallopian tube that may underlay all the various subtypes of ovarian carcinoma. It has also been argued that the distal fallopian tube represents a developmental “transition zone,” analogous to that present in the cervix, which is prone to malignant transformation [42].

Notwithstanding the convincing evidence that the fallopian tube is the major site of origin for HGSOC, it remains established that ovulation is a consistent risk factor in epidemiological studies. To explain this, it has been proposed that the proximity of the fimbriae to the ovarian surface might subject it to many of the same pro-inflammatory mediators and ROS thought to contribute to the development of genotoxic stress in the OSE following ovulation [37]. Yet despite the most diligent examination, a significant percentage of HGSOC cases present without fallopian tube involvement [15]. This has led some to suggest that there still might be precursor cells in the ovary that underlie such cases [15]. A new unifying theory contends that these cases arise from the early implantation of FTSECs in the OSE through a process called “endosalpingiosis” [42]. In this scenario, the fallopian tube epithelium might become incorporated into the same CICs that are the preferred site of origin for HGSOC in the ovary [40,42]. As has already been highlighted, the microenvironment of the ovary is more favourable for inducing neoplastic transformation compared to that of the fallopian tube [37,42]. Although endosalpingiosis has been demonstrated in mouse ovaries, the same process has never been observed in humans [42]; thus, the precise progenitor for a substantial percentage of HGSOC cases remains obscure.

## 6. Dissemination

High-grade serous ovarian carcinoma notably does not require the blood or lymph in order to metastasize. For most other epithelial cancers to spread, tumour cells must typically undergo a sequence of cellular transformations to traverse the basement membrane, migrate to and invade the vasculature, survive in suspension, extravasate and re-establish themselves as a colony at a distant site. By contrast, HGSOC typically spreads by direct extension to the adjacent organs within the peritoneal cavity or through the detachment of cells from the primary tumour [11]. For a tumour growing on the surface of the ovary or fallopian tube, there are no anatomical barriers capable of restricting the spread of tumour cells throughout this fluid-filled space between the body’s visceral organs [56]. Once the cells have exfoliated from the primary tumours site, either singly or in clusters, they become suspended in the peritoneal fluid and are spread by a passive process that follows the physiological flow of this fluid around the peritoneal cavity [11]. These cells then can implant and seed distant organs or tissues with nests of cancer cells, which develop rapidly into secondary tumour nodules.

Although virtually every organ or structure within the peritoneal cavity may be involved in secondary dissemination, HGSOC cells are known to exhibit a particular predilection for the omentum [11]. In fact, 80% of patients with HGSOC present with omental metastases [57]. Composed largely of energy-dense adipocytes, this large fat-pad extends from the stomach and covers the intestines. It has been hypothesized that HGSOC preference for the omentum stems from a cellular metabolic requirement for fatty-acid-based catabolism (β-oxidation) [57]. It has been shown that adipocytes produce pro-inflammatory cytokines such as IL-8 that promote the homing and invasion of tumour cells [57]. In the same study, co-culturing adipocytes with ovarian cancer cells was seen to promote greater lipolysis in adipocytes and β-oxidation in cancer cells [57]. This co-culturing also led to increased proliferation of ovarian cancer cells in-vitro and rapid growth of transplanted tumours in-vivo [57].

While HGSOC spreads readily within the peritoneal cavity, its metastatic growths only invade the surface of affected organs [11]. Secondary tumours typically colonize the mesothelial cell layer but invade no further, leaving the deeper lamina largely intact [11]. Spread outside the peritoneal cavity is uncommon, although certain pelvic and/or para-aortic lymph nodes sometimes can be involved [58].

Hematogenous spread is thought to be largely precluded, based on the observation that patients treated with peritoneovenous shunts—having received a large number of tumour cells into the circulation—mostly failed to develop any distant metastases even two years after the procedure [59]. There is the potential, however, for metastasis to the liver, while in the most advanced stage of HGSOC tumour cells may also cross the diaphragmatic barrier and enter the pleural space, where they can cause pleural effusions or even implant in the parenchyma of the lung [8].

Patients with late-stage disease frequently develop ascites featuring a prominent cellular component, which are commonly referred to as “malignant ascites”. HGSOC cells might participate in the formation of these ascites either by blocking the lymphatic drainage or by secreting vasoactive and angiogenic factors which promote vascular permeability [11]. A lingering mystery is the role of multicellular structures in the pathogenesis of HGSOC. These structures frequently appear in the form of spheroids or aggregates of suspended tumour cells and commonly are isolated from the ascites of patients with advanced disease. They have been proposed to represent a fundamental unit of metastatic spread, while also forming a chemo-resistant niche to allow for the HGSOC cancer cells to survive therapy [11]. Importantly, the formation of multicellular structures might allow the cells to survive in anchorage-independent conditions by preventing anoikis [11].

## 7. Symptomatology, Diagnosis and Staging

One of the principal factors influencing the elevated mortality of HGSOC patients is the inability to diagnose the disease at an early, localized stage. Only about 13% of cases of serous ovarian carcinoma are diagnosed at stage I or stage II [60]. In fact, the vast majority of cases usually are diagnosed at the stage of distant metastasis, which greatly prejudices an individual’s prognosis [4]. The 10-year survival of patients diagnosed with early-stage HGSOC is 55%, compared to only 15% for those having presented with an advanced-stage disease [60].

There are currently no effective screening strategies for the early detection of ovarian cancer [8]. A recent trial evaluating the utility of transvaginal ultrasonography in combination with serum CA125 levels demonstrated some promise in terms of early detection but failed to improve patient outcomes [61,62]. Genetic tests might be useful to detect heritable BRCA mutations in patients with a known family history of breast and ovarian cancer [8]. In such cases, the at-risk individual might elect to undergo risk-reducing prophylactic surgery such as bilateral salpingo-oophorectomy, typically upon completion of childrearing or by the age of 40 at the latest [22]. This technique has proven to be effective in preventing the emergence of ovarian cancer in as much as 85–90% of cases [63]. Finally, a very recent study shows promise in the early detection of ovarian cancers based on the genetic analysis of mutations detected in the DNA recovered from liquid biopsies obtained during routine Papanicolaou tests [64].

Because the symptoms associated with HGSOC are often diverse and non-specific, there is usually little likelihood that a patient will encounter the appropriate medical specialist in time for an early diagnosis to be made [8,65]. Symptoms typically are gastrointestinal and include abdominal pain, bloating, nausea, constipation, anorexia, diarrhoea and acid reflux [8,9]. Other symptoms include fatigue, back pain, tenesmus, as well as elevated urinary frequency [8,9]. At an advanced stage, respiratory symptoms might be present such as cough and dyspnoea [9] (Figure 4C).

Unfortunately, by the time a patient becomes symptomatic her disease is found to be at an advanced stage between 75–80% of cases [9]. This differs from other forms of EOC such as clear-cell carcinoma that typically become symptomatic at a far earlier stage [8]. If a diagnosis of EOC is suspected, the patient will typically be subjected to a pelvic and rectovaginal examination along with radiographic imaging such as transvaginal or abdominal ultrasonography, CT, MRI or PET [8]. Blood levels of CA125 also might be measured, which in combination with other tests, might be of diagnostic value [8]. Imaging will typically reveal complex, hyper-vascular pelvic masses and omental/peritoneal nodules [9]. Serum CA125 levels often are elevated, especially in advanced cases, with average values that can range between 500–1000 U/mL [66]. Advanced disease typically will feature extensive peritoneal carcinomatosis, involving most of the major abdominal organs and may be associated with the accumulation of large volumes of ascites [11].

To aid in diagnosis, laparoscopic surgery usually is performed to obtain a tumour sample for biopsy and to aid in the staging of the disease [8]. The most recent (2014) FIGO staging system is based on the degree of dissemination of the disease at diagnosis. At stage I, the cancer still is confined to the ovaries or fallopian tubes [67]. By stage II, the disease already has spread to other pelvic organs such as the uterus [67]. Stage III involves spread beyond the pelvis to organs or tissues within the peritoneal cavity or to the retroperitoneal lymph nodes [67]. Stage IV results from spread beyond the peritoneal cavity, including to the lungs and involvement of inguinal and other extra-abdominal lymph nodes [67]. The end stage of the disease is characterized by malignant bowel obstruction due to the formation of fibrous adhesions between loops of the bowel by the metastatic tumours [56]. This impedes the patient from normal alimentation, leading to cachexia, malnutrition, and, eventually, death from factors which may include intercurrent infection [56].

## 8. Genetics

A major milestone in the understanding of HGSOC occurred as the result of a 2011 study by the cancer genome atlas (TCGA) network which sought to evaluate the disease’s genetic features through whole exome sequencing of samples from 316 patients [68]. This revealed that the genomic landscape of HGSOC is characterized by profound genomic instability with few recurrent gene mutations other than in TP53 [68]. This was in contrast to Type-1 EOCs which are characterized by frequent oncogenic mutations in genes such as BRAF, KRAS, PTEN, CTNNB1 and PIK3CA while being P53 wild type [68]. In the case of HGSOC, it was found that upwards of 96% of the samples contained somatic TP53 mutations, seemingly suggesting that this mutation is a defining feature of HGSOC and one likely to be required for disease initiation [22]. Retrospective studies established that the small percentage of P53 wild type samples from the aforementioned TCGA study derived from patients whose disease had likely been misdiagnosed as HGSOC [69]. Thus, one might conclude that TP53 mutations are virtually ubiquitous in HGSOC. Because P53 commonly is found to be mutated even in the STIC precursor lesions, it is likely that this event is one of the earliest in the sequence of carcinogenesis for HGSOC [22].

Further studies have shed light on the precise nature and function of the P53 mutations present in patients with HGSOC. One study, using data from the International Agency for Research on Cancer (IARC) P53 database, reported that 70.4% of TP53 mutations were in fact missense mutations, which encode for a protein with an amino-acid substitution [70]. There was a much smaller contribution from frameshift, nonsense and splice mutations, which affect 12%, 8.67% and 5.1% of patients [70]. These mutations encode for truncated or malformed proteins, which are equally likely to confer a total abolition of function. The missense mutations can result in three distinct phenotypes depending on their effects on P53 protein function: there is the potential for either a loss of function, dominant negative or a gain of function mutation [70].

The P53 protein consists of multiple structural domains, each specifying one aspect of the protein’s functional behaviour. Around 80% of total mutations are located in the central DNA binding domain of the P53 protein. These likely result in a loss of function due to the inability to serve as a transcription factor to modulate the expression of target genes [70]. Because P53 functions as a tetramer, many missense mutations also may result in the formation of a dominant-negative protein which can inhibit tetramerization, even in the presence of residual P53 wild type protein [70]. Mutant p53 protein is far more stable in the cell than its wild type counterpart due to the inability to interact with its inhibitor HDM2, which normally ensures its timely degradation by the proteasome [70]. This increased stability and thus, the higher protein levels of mutant P53, may enable it to possess an additional, oncogenic gain of function activity [70]. Studies have shown that the type and location of a patient’s TP53 mutation have implications on the individual’s prognosis [71,72].

Besides TP53, few other gene mutations are common between patients with HGSOC. The TCGA study found BRCA1 mutations in about 12.5% of patients (9% germline mutations and 3.5% somatic mutations), BRCA2 mutations in about 11.5% of cases (8% germline mutations and 3.3% somatic mutations) and a smaller number of mutations involving CSMD3 (6%), NF1 (4%), CDK12 (3%), GABRA6 (2%) and RB1 (2%) [68]. By contrast, there appears to be a much more prominent role for gene copy-number variation in HGSOC due to genomic instability, resulting in the amplification or loss of many genes. The most frequent focal amplifications involve the genes CCNE1, MYC and MECOM. Each is involved in more than 20% of the samples analysed [68].

An analysis integrating mutational frequency, copy number alterations and changes in gene expression has provided evidence of the main pathways involved in HGSOC pathogenesis. This method revealed that the homologous recombination pathway of DNA repair is defective in 51% of cases [68]. This involved mutations to BRCA1 and BRCA2 (germline and somatic) in 20% of patients, with a further 11% having BRCA1 silencing by DNA hypermethylation [68]. In patients with hereditary BRCA1/2 mutations, the other allele almost always displays loss of heterozygosity (LOH) [22]. One study determined that 91% patients with BRCA1 germline mutation displayed locus specific LOH compared to 72% of non-carriers [73]. Another group estimated that 100% of patients with germline BRCA1 mutations have LOH along 76% of those with inherited mutations in BRCA2 [74]. Moreover, the type of mutation present may be significant, especially in the case of BRCA2 for which patients with truncating mutations in the RAD15 binding domain had a survival advantage over individuals with other mutations in the same gene [75]. Other genes encoding proteins in the homologous recombination pathway are affected recurrently in HGSOC, including PTEN, RAD51C, ATM and ATR, as well as many of the Fanconi anaemia genes [68]. The involvement of these genes would suggest that defects in homologous recombination DNA repair play a major role in the aetiology of HGSOC. Intriguingly, CCNE1-amplification cases segregated from those with BRCA mutation, suggesting that there could be two distinct pathways driving the pathogenesis of HGSOC with a heterogeneity among patients [68]. BRCA-mutant patients notably possessed a significant overall survival advantage over those with CCNE1 amplification [68]. In general, it has been seen that patients with BRCA mutations initially respond better to chemotherapy and therefore have a better 5-year survival than non-mutant individuals [76]. This survival advantage, however, is not maintained in the long-term in the case of BRCA1 but persists with a small advantage in the case of BRCA2 [76].

Other pathways frequently altered include: RB1 (67%), PI3K/Ras (45%) and NOTCH (22%) [68]. The TCGA study also identified activation of the FOXM1 transcription factor pathway as another important hallmark of HGSOC, with 87% of patients presenting with overactivation of the transcription network downstream of this protein [68]. Importantly, FOXM1 normally is suppressed by P53 in the context of DNA damage, suggesting that P53 mutation might contribute to the overactivity of this pathway [68].

Many studies have highlighted the important role of PI3K-AKT pathway in HGSOC [22]. The TCGA study linked amplifications of PIK3CA, PIK3CB and PIK3K4 with decreased overall survival [68]. Another study found that, in patients with advanced HGSOC, PI3KCA mutations were found in 5% of samples, along with amplification of PI3KCA and AKT2 in 12% and 10% of samples [77]. It also has been argued that most genetic studies underestimate the percentage of cases with the loss of expression of PTEN [78]. Using an immunohistochemical analysis by tissue microarray, one study showed that around 50–75% of cases are characterized by PTEN loss or under-expression [78]. Also, GAB2, an adaptor protein between the Ras-MAPK and PI3K-AKT signalling pathways, was shown by one study to be amplified recurrently in 44% of cases [79].

Analysis of haploinsufficiency has suggested that the autophagic pathway also is significantly disrupted through gene deletion [80]. One study reported that the genes BENC1 and LC3 were mono-allelically deleted in 94% of patients with HGSOC [80]. In addition, it was shown that HGSOC cell lines were hypersensitive to treatment in-vitro with autophagy inhibitors [80].

The downregulation of the Let-7 family of micro-RNA also is thought to play an important role in the aetiology of HGSOC, leading to the translational overexpression of many proteins, including the DNA-binding factor HMGA2 [81]. This protein is an important regulator of chromatin conformation and functions as a structural factor regulating the assembly of the enhanceosome complex, thus participating in the expression of many genes. HMGA2 was found to be overexpressed in 64% of HGSOC tumours by immunohistochemistry and its expression correlated with a less-differentiated phenotype and poor patient prognosis [81,82].

Although genomic instability is a feature of HGSOC, only a few recurrent recombination events have been identified to result in the creation of fusion genes [22]. One such event is the formation of the inter-chromosomal fusion gene CDKN2D-WDFY2, which was detected in 20% of cases of HGSOC [83]. CDKN2D (a.k.a P19) is a cell-cycle specific regulator of AKT signalling and the fusion protein was demonstrated to be sufficient to activate the PI3K-AKT pathway in transfected cells [83].

## 9. Gene Expression Profiling and Molecular Subtypes

Although referred to as a singular class of malignancy, a recent line of evidence from studies employing gene expression profiling has revealed that HGSOC actually is characterized by a whole spectrum of molecular diversity. One such influential study, by the group of Tothill et al., succeeded in delineating four distinct molecular subtypes of HGSOC with significant correlations to patient outcome [84]. Using 285 predominantly high-grade serous tumour samples, their analysis of differential gene expression segregated the pooled data into 6 robust clusters, which were accorded the names C1-C6 [84]. Of these, clusters C3 and C6 were deemed unlikely to represent HGSOC [84]. Cluster C1 is marked by its association with a reactive stromal signature and the upregulation of genes associated with extracellular matrix production/remodelling, cell adhesion, cell signalling and angiogenesis [22,84]. At the histopathological level, this subtype is distinguished by extensive myofibroblast infiltration (desmoplasia) [22,84]. It was discovered that this signature was associated with a poor overall prognosis [84]. By contrast, the C2 was termed “immunoreactive” because of its association with higher numbers of tumour-infiltrating CD3^+^ T-lymphocytes and a gene expression signature defined by the upregulation of genes involved in immune cell activation [22,84]. This subtype was found to have a greater overall survival [84]. C4 demonstrated a low stromal response with certain parallels in gene expression to C2 but with elevated CA125 [22,84]. It also was associated with a better prognosis [84]. C5 exhibited a signature that featured many genes involved in mesenchymal development, including certain HOX genes, high-mobility group members, as well as WNT/catenin and cadherin signalling pathways [22,84]. This group also was found to have an inferior overall survival [84].

A subsequent study by the TCGA group built upon these results, using a refined methodology to identify 4 overlapping subtypes which were named immunoreactive, differentiated, proliferative and mesenchymal [68]. This study, however, failed to identify any significant correlations to patient outcomes [22]. Conversely, a recent re-analysis of the TCGA dataset, using a more sophisticated protocol, managed to elaborate on the molecular signature of the 4 previously identified subtypes and to demonstrate prognostic significance for each [85]. The mesenchymal and proliferative subtypes possessed the worst overall survival, while the immunoreactive featured a better prognosis, with the differentiated being intermediate on the scale [85]. Another group later confirmed these results [86]. Recently, due to persistent heterogeneity in the differentiated subtype, it was decided to declare a further group known as anti-mesenchymal due to the downregulation of genes involved in the mesenchymal subtype [87]. This new subtype was associated with a better prognosis [87]. The popularity of these molecular subtypes has instigated efforts to introduce a new histopathological classification system based on characteristics relating to each of the molecular subtypes [22,88].

Many groups are publishing reports on the prognostic significance of certain gene signatures [22]. In some cases, these might involve hundreds of genes, while in others only a few. In one instance, the expression of certain genes involved in DNA repair pathways has been associated with a favourable prognosis after platinum chemotherapy [89]. Another identified the expression of HIF-1α and its associated response genes as predictive of poor overall survival [90]. Yet another linked genes encoding extracellular matrix proteins involved in collagen remodelling with poor overall patient survival and high metastatic potential [91]. Efforts also have sought to define signatures predicting the extent of optimal debulking after surgery or the response to platinum chemotherapy or PARP inhibitors [90,92,93].

## 10. Surgery

The primary recourse initiated in patients with HGSOC is a procedure called surgical cytoreduction or “debulking.” The goal of this surgical approach is to achieve macroscopic total resection of all the disseminated tumour masses contained within the peritoneal cavity of the patient [8]. The surgery is typically the responsibility of a gynaecological oncologist, although these may not always be available, which often negatively impacts the quality of treatment and patient outcomes [8]. The extent and difficulty of the procedure is directly proportional to the disease stage. Advanced patients have a diminished likelihood of operative success due to the widespread nature of the many metastatic foci, which often prevents complete cytoreduction [94]. The aggressive surgical technique involves the en bloc removal of all gross tumour tissue, the reproductive organs and the sigmoid colon along with a complete peritonectomy and omentectomy [8,11]. Systematic dissection of the pelvic and para-aortic lymph nodes also is usually performed depending on the stage of the patient and degree of nodal involvement [8]. A successful surgical outcome is defined as one resulting in the absence of any macroscopic residual disease [8]. In practice, however, the optimal degree of cytoreduction is identified as one resulting in less than 1 cm residual cancer [8]. Anything above this is considered to be a suboptimal result.

The level of primary cytoreduction achieved is perhaps the most important prognostic factor influencing the eventual fate of the patient [9]. In cases where total resection has been accomplished, the long-term outlook is comparatively favourable with some patients even being cured after subsequent chemotherapy [8]. The outcomes for patients with “optimal” cytoreduction are substantially worse than those with no residual disease but nevertheless better than those with suboptimal cytoreduction [95,96,97]. Because of its distinctive pathobiology, HGSOC is one of the few epithelial cancers in which the removal of metastatic tumours has been found to improve overall survival [11].

## 11. Cytotoxic Chemotherapy

Following successful cytoreductive surgery, virtually all patients with HGSOC are recommended to undergo adjuvant chemotherapy [8]. This contrasts with other, primarily low-grade, subtypes of ovarian cancer where the extent of treatment is dictated by the disease stage, with many patients diagnosed with localized (stage I) disease deemed not to require any further treatment after surgery [8].

**Figure 5:**
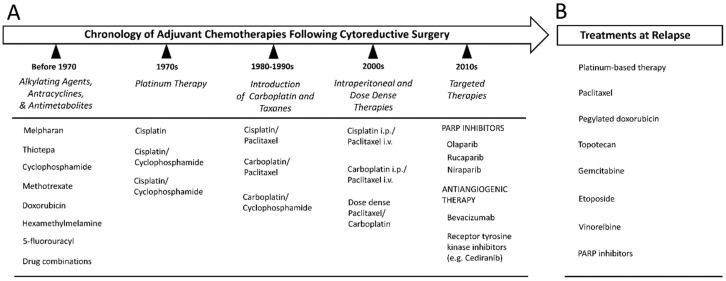
Historical perspectives of the treatment of high-grade serous ovarian cancer. (**A**) Evolution of chemotherapies; (**B**) salvage therapies for recurrent disease.

The type of chemotherapy regimen received by the patient is the same irrespective of the EOC subtype involved [98]. Historically, ovarian cancer was one of the first malignancies to be successfully treated with cytotoxic chemotherapy (Figure 5A) [99]. The first class of chemotherapeutic drugs to be developed were the alkylating agents, which were introduced in the 1950s [100]. These function as antineoplastic agents through their capacity to cause DNA damage via the addition of bulky alkyl groups to guanine nucleotide bases. The effect is usually sufficient to inhibit proper DNA synthesis. Many such agents were previously used in the treatment of ovarian cancer including melphalan, thiotepa and cyclophosphamide [99]. They were joined in the clinical setting by other types of cytotoxic agents including methotrexate, 5-fluorouracil, doxorubicin and hexamethylmelamine [100].

Although many of these drugs demonstrated good single-agent activities in the treatment of ovarian cancer, it was ascertained promptly that the most effective strategy would be to employ these agents in combination [100,101]. This was based on the theory that multiple drugs, each with different mechanisms of action, would behave synergistically and reduce the risk of the disease acquiring chemoresistance [99]. In the 1970s, many such combinations were in use for the treatment of advanced ovarian cancer, with the most popular regimens consisting of cyclophosphamide and doxorubicin, along with methotrexate and 5-fluorouracil [100].

Since the late 1970s, interest has settled around the use of platinating agents for the treatment of this disease, so-much-so that the therapeutic standard-of-care for ovarian cancer in recent decades has been referred to as “platinum-based therapy.” The first such drug to be approved for clinical use was called cisplatin. Its introduction followed the results of trials that validated its effectiveness in the context of recurrent disease resistant to alkylating agents and doxorubicin [100]. It was incorporated into primary chemotherapeutic regimens either singly or in combination with cyclophosphamide, doxorubicin or hexamethylmelamine, among others [100].

The late 1980s saw the introduction of a new platinating agent in the form of carboplatin. A set of trials, completed in 1992, concluded that carboplatin demonstrated comparable effectiveness to cisplatin in the treatment of ovarian cancer in combination with cyclophosphamide but with a far more favourable toxicity profile [102,103]. These results prompted most clinicians in developed nations to begin replacing cisplatin-based regimens with those utilizing carboplatin [100].

The late 1980s also witnessed the development of a new class of drugs, the taxanes, which promptly began testing in the context of ovarian cancer. These drugs, of which paclitaxel is the prototype, were first isolated from the bark of the pacific yew tree (Taxus brevifolia) and function by inhibiting tubulin depolymerization. This stabilization of the microtubular cytoskeleton results in dysregulation of the cell cycle, culminating in mitotic failure and cell death [104]. Early reports indicated that paclitaxel could achieve objective responses in women with advanced, platinum-resistant ovarian cancer [100,105,106].

In 1996, a landmark clinical trial evaluated the effectiveness of cisplatin-paclitaxel combination therapy versus cisplatin-cyclophosphamide. Results indicated that cisplatin-paclitaxel combination therapy was capable of significantly improving objective response rates, progression free survival and overall survival, compared to the then standard regimen of cisplatin-cyclophosphamide [107]. In the interest of averting the toxic side-effects of cisplatin, subsequent trials have confirmed similar results using carboplatin instead of cisplatin along with paclitaxel [108,109,110,111]. This combination has, for the last 20 years, been the standard of care for the treatment of ovarian cancer [100].

Efforts in recent years have centred rather on optimizing the current platinum-taxane treatment regimen. The usage of a dose-dense treatment schedule for paclitaxel (weekly) in combination with carboplatin (every 3 weeks) was associated with improvements in outcomes for Japanese women [112]. A similar trial in Western women (MITO7) failed to show any differences in outcome for a dose-dense schedule, although the aims and design of the study were different and thus complicate the interpretation of the results [113]. In another recent trial, entitled GOG 262, patients were administered a regimen of carboplatin plus either a dose-dense (weekly) or a standard (every 3 weeks) treatment schedule of paclitaxel, with or without bevacizumab [114]. In the minor cohort that did not elect to receive bevacizumab, it was reported that the dose-dense (weekly) schedule was associated with an improvement in progression-free survival but only by 3.9 months [114].

Another current approach is intraperitoneal delivery of the chemotherapeutic agents. The rationale behind this method is that, because ovarian cancer is almost always confined to the peritoneal cavity, the delivery of the drugs directly to this environment might achieve greater local drug concentrations [100]. Studies indeed have shown that intraperitoneal delivery can achieve 20-fold increase in cisplatin concentration and 1000-fold increase in the local concentration of paclitaxel [115,116]. This increase in local concentration would be highly dependent upon the size of the residual disease present, since the depth of penetration of cisplatin and other agents into tumour tissue is thought to be limited to only a few millimetres from the surface of the peritoneal cavity [100]. Three trials have shown improvements in progression-free and overall survival for patients treated with intraperitoneal cisplatin with intravenous paclitaxel [117,118,119]. These results convinced the National Cancer Institute of the United States (NCI), in 2006, to notify physicians that intraperitoneal cisplatin treatment improves patient survival [120]. Nevertheless, intraperitoneal chemotherapy has not been adopted widely in the clinical setting due to elevated toxicity and poor patient tolerability [94].

The administration of carboplatin via the intraperitoneal route is currently being explored. One major trial (GOG 252) reported that it was better tolerated than intraperitoneal cisplatin but failed to show any meaningful survival benefit of the intraperitoneal approach, unlike the previous trials (many confounding factors) [120,121,122].

The current intravenous treatment protocol consists of 75 mg/m^2^ cisplatin infusion, plus 135 mg/m^2^ paclitaxel infused over 24 h every 3 weeks for a total of 6 cycles [100]. The usage of cisplatin requires aggressive rehydration to prevent nephrotoxicity [100]. For carboplatin, the dose is area under the curve (AUC) equal 6 along with paclitaxel 175 mg/m^2^ infused over 3 h with the same treatment schedule and number of cycles [100].

Patients that are too ill to undergo initial surgical cytoreduction or whose disease is too extensive to allow for complete resection, may choose to receive neoadjuvant chemotherapy (NACT) [8]. These individuals are administered the first 3 cycles of chemotherapy, which are then followed by an interval whereupon patients undergo surgical cytoreduction and finally the remaining 3 rounds of chemotherapy [8]. Two randomized trials have concluded that NACT is not inferior to initial surgery in terms of progression-free survival and overall survival [123,124].

## 12. Relapse and Treatment Resistance

Although 70% of ovarian cancer patients initially respond favorably to the first application of platinum-based chemotherapy [56], it is estimated that ≥80% of these will eventually relapse at some stage [8]. For the subset of patients whose disease is judged to be refractory to the front-line chemotherapy, alternative or second-line drug combinations may be utilized in an attempt to elicit an objective response [100]. After successful completion of chemotherapy, the patient is typically assessed radiologically or using CA125 as a biomarker of disease activity level [94]. In at least half of patients, residual cancer cannot be detected using imaging studies and serum markers after 5 months of treatment [56]. During remission, the patient is typically followed-up every 2–4 months with a physical examination or optional radiographic imaging and serum CA125 bioassay [8]. Recurrence generally is asymptomatic at first and frequently is detected by an increase in CA125 levels [8]. The doubling of such levels above the upper limit of normal is considered to be the threshold for diagnosing recurrence [94]. Rarely, a CT scan might detect an asymptomatic recurrence or the relapse might present with symptoms and a clinically-detectable mass [94].

Although CA125 has proven useful for detecting early recurrence, treatment typically is not re-introduced in the absence of clinical symptoms [8]. One study established that early re-treatment was not associated with any improvements in patient outcome [125]. If relapse consists of a discrete, highly localized tumor mass (rare in the case of HGSOC), then a second cytoreductive surgery may be performed, although a recent meta-analysis of patient data found no benefit to this approach in terms of prolonging survival [8,126]. Otherwise, surgery is only used to palliate the effects of intestinal obstruction associated with an isolated site of disease [94].

Typically, patients are re-treated with the standard platinum-based chemotherapeutic regimen [8]. The decision to re-use platinum is complicated by the presence of persistent side-effects from previous treatment, such as neuropathy and pancytopenia, as well as the potential for life-threatening platinum hypersensitivity reactions [8]. Approximately 50% of patients possess recurrent disease that is still responsive to re-treatment with platinum, albeit with diminishing returns as the progression-free survival invariably decreases with each successive platinum therapy [8].

In patients relapsing with a disease that is platinum-resistant, a variety of alternative treatment modalities may be pursued, including the use of pegylated liposomal doxorubicin, topotecan, gemcitabine, etoposide and vinorelbine (Figure 5B) [8]. The average response rate to this kind of salvage therapy is only about 10–15% with a median progression-free survival of 3–4 months [8]. Ultimately, 80–90% of patients diagnosed with advanced-stage disease will develop treatment resistance, which inevitably heralds eventual mortality [15].

## 13. Targeted Therapies

The ideal form of cancer chemotherapy would involve targeting only those pathways known to be abnormally activated in the context of the cancer cell, while sparing the cytotoxic effect from the body’s normal cells. This is the philosophy behind targeted therapy, which has ushered in a new era in the way many cancers are treated. Sadly, in relation to ovarian cancer, this approach has not been especially fruitful as of yet, with only a few new treatments reaching the clinic and yielding only marginal improvements in outcome (Figure 5A). This is partly related to the molecular biology of HGSOC which does not often present with many oncogenic alterations that can be targeted easily with small-molecular inhibitors [15]. That being said, a number of avenues are being explored currently in order to develop novel therapeutics for this disease.

One of the more promising targeted approaches relates to a class of drugs known as PARP inhibitors. HGSOC is characterized by widespread genomic instability and the majority of patients possess some deficiency in DNA repair pathways (germline or somatic), particularly those involving the repair of DNA double-strand breaks by homologous recombination. The proteins encoded by BRCA1 and BRCA2 are involved in this pathway along with many others. In patients with a deficiency in homologous recombination, the cancer cells are over-reliant on the poly (ADP-Ribose) polymerase (PARP) mediated base excision repair (BER) of single-strand breaks to resolve spontaneous DNA damage [127]. As such, drugs targeting PARP would be expected to display significant anti-tumor activity in these patients due to synthetic lethality [127]. This term denotes the phenomenon whereby the loss of function in one gene can be tolerated by a cell but can become lethal when combined with the loss of an additional gene product or pathway [128]. Drugs exploiting this concept are considered desirable because they would have a much greater specificity for tumor cells due to the inherent requirement for a further underlying mutation in order to be effective [128].

The first PARP inhibitor to be tested in patients with HGSOC was olaparib [127]. Its use has primarily been envisaged as a treatment for recurrent disease or as a maintenance therapy to prolong progression-free survival [8]. Early phase I and randomized clinical trials of olaparib showed an impressive clinical response in patients with recurrent HGSOC with BRCA mutations [94]. In one phase I trial, a 28% radiologic response was observed in patients treated with olaparib 200 mg twice daily [129]. A subsequent phase II trial established that olaparib was significantly more effective in relapsed BRCA mutant patients than pegylated liposomal doxorubicin [130]. Another phase II trial using a 400 mg dose of olaparib twice daily, in both BRCA mutant and wild type recurrent HGSOC, showed objective response rates of 50% in the BRCA-wild-type cohort and 60% in the BRCA-mutant cohort for those whose disease was platinum-sensitive [131]. In patients with platinum-resistant disease, the response rates were 33% in the mutation-positive cohort but only 4% in the BRCA-wild-type cohort [131]. A trial testing olaparib as a maintenance therapy for patients with relapsed disease showed a significant increase in progression-free survival compared to placebo but without any increase in overall survival [132]. This study also demonstrated that some patients without BRCA mutations also could benefit from this type of treatment [22,133]. In 2014, the European Medicines Agency (EMA) approved olaparib for use as a maintenance therapy in cases of platinum-sensitive recurrent disease in patients with BRCA mutations [8]. The FDA also approved its use as a monotherapy for patients with germline BRCA mutations who already have undergone 3 prior applications of chemotherapy, regardless of platinum sensitivity [8].

A phase III study (SOLO2) confirmed the efficacy of olaparib as a maintenance therapy in BRCA-mutant individuals with relapsed platinum-sensitive disease, though the effect on the non-mutant population was not evaluated [134]. Olaparib also has been tested as a supplement to traditional platinum-based therapy in the context of platinum-sensitive, relapsed disease [127]. One phase II trial established that progression-free survival was longer in the cohort receiving olaparib after carboplatin-paclitaxel therapy but with the effect being greater for BRCA mutant patients [135]. There was, however, no difference in overall survival and toxicity was greater in the group receiving olaparib [127,135].

Recently, the FDA approved the use of two other PARP inhibitors, rucaparib and niraparib, to treat patients with relapsed ovarian cancer, irrespective of BRCA-mutation status or platinum-sensitivity [136]. This was based on the outcome of two Phase-III clinical trials showing the effectiveness of these drugs in prolonging progression-free survival in both BRCA-mutant and wild-type individuals when administered as a maintenance therapy in patients with platinum-sensitive, recurrent disease [137,138].

Another therapy being investigated involves targeting the tumor micro-environment through the use of anti-angiogenic agents such as bevacizumab. This humanized monoclonal antibody targets the cytokine VEGF-A, which directs the recruitment of blood vessels to the tumor, something required for its growth beyond a certain size [94]. Two important clinical trials (ICON7 and GOG 218) showed an increase in progression-free survival with the addition of bevacizumab to the standard carboplatin-paclitaxel regimen as maintenance therapy [139,140]. This prompted the approval of this drug in Europe for use as a maintenance therapy [8]. Two other trials in the context of platinum-sensitive [141] and platinum-resistant [142] disease have shown that the addition of bevacizumab to cytotoxic chemotherapy improves progression-free survival. The use of bevacizumab in the context of advanced EOC carries the risk of significant adverse effects, including gastrointestinal perforation or fistula, hypertension, proteinuria, neutropenia and wound disruption [8,98].

Other anti-angiogenic therapies aim to inhibit the VEGF receptor and other related receptor tyrosine kinases (RTK) [8]. One such agent is pazopanib. In one trial, the use of this agent as a maintenance therapy significantly prolonged progression-free survival, albeit without increasing overall survival and with a significant toxicity profile [143]. Other anti-angiogenic agents currently are being tested, including nintedanib, trebananib, sunitinib, cabozantinib and cediranib [8]. The most promising of these is probably cediranib, which has been shown to exert significant single-agent activity in the context of both platinum-sensitive and resistant-relapsed disease [144,145]. It also has been shown to increase progression-free survival when used in combination with cytotoxic chemotherapy or as a maintenance therapy [146]. A retrospective assessment concluded that the use of cediranib as a maintenance therapy was not associated with a reduction in patient quality of life after 1 year of use [147]. One recent phase II study has explored a combination treatment of cediranib with olaparib in patients with platinum-sensitive, relapsed ovarian cancer [148]. The combination showed a significant increase in progression-free survival compared to olaparib monotherapy, though this effect was seen only in BRCA wild-type individuals [149]. BRCA germline-mutant patients, whose response to olaparib alone is greater than in wild-type individuals, had no further improvement with the addition of cediranib [22,149].

Another potential targeted therapy is the inhibition of AKT signaling. One recent phase Ib/II study reported promising clinical activity of an oral AKT inhibitor (afuresertib) in combination with carboplatin and paclitaxel in platinum-resistant ovarian cancer [15,150].

## 14. Preclinical Models of Study

### 14.1. In-Vitro Models

The use of cancer cell lines to model HGSOC in-vitro is perhaps the most frequently employed method of interrogating the disease experimentally. It is thus of paramount importance to ensure that the cell lines in current usage accurately represent the disease’s fundamental biology, while maintaining a practical ease of use and accessibility to all researchers for the purpose of standardization. These cell lines should each possess a well-annotated source of origin, with available information relating to the clinical history of the donor patient and the precise manner in which they were initially established. Unfortunately, until recently, most of the cell lines in widespread usage were poorly characterized, with obscure origins and uncertain histopathology [151]. These included many of the top cell lines by publication figures including: SK-OV-3, A2780, OVCAR-3, CAOV-3 and IGROV-1 [151]. These cell lines, including the most commonly used ovarian cancer cell lines SK-OV-3 and A2780, were popular because they rapidly and reproducibly form discrete tumors when injected orthotopically or subcutaneously into nude mice, while also being manipulated easily using transfection techniques [152]. The publication by the TCGA, in 2011, of a comprehensive genomic characterization of HGSOC finally would permit a determination on the suitability of the then popular cell lines to be made on the basis of their genetic similarity to the disease [68]. To this end, Domcke et al. compared the genomic features of the cell lines purported to represent HGSOC with the data derived from the TCGA study of primary tumor samples [151]. This yielded a number of shocking results, in that virtually all the cell lines commonly used in the literature as models of HGSOC were deemed, in fact, to poorly recapitulate the genetic features of the disease. This was especially true for the two most popular cell lines, SK-OV-3 and A2780, which were not assigned a histological subtype by the originator but which together contributed to about 60% of all publications using ovarian cancer cell lines [151]. While almost 100% of HGSOC cases are characterized by TP53 mutation, these two cell lines are P53 wild-type and contain very few gene copy number alterations [151]. Since then, a variant of SK-OV-3 has been identified that contains a P53 deletion but this is far more likely to have emerged in culture [152]. These cell lines feature many of the mutations typically found in the other histotypes of EOC including ARID1A, BRAF, PIK3CA and PTEN [151]. Based on this evidence it is possible to classify both A2780 and SK-OV-3 as being derived from Type-1 non-serous tumors and thus unrelated to HGSOC [151]. IGROV-1, another popular cell line quoted as being from HGSOC, was P53-mutant but displayed a hypermutant phenotype, which is not among the features of this disease [151]. Clustering analysis grouped IGROV-1 among the endometriosis-derived subtypes (clear-cell and endometrioid) [151]. Other cell lines, such as OVCAR-3, possessed a similar mutational profile to HGSOC but had copy-number alterations (CNAs) that diverged from the TCGA tumor-sample mean [151]. These cell lines probably were derived from cases of HGSOC but do not recapitulate perfectly the genomic features of the disease.

The cell lines ascertained to best fit the genomic picture of HGSOC were Kuramochi, OVSAHO, SNU119, COV362 and OVCAR-4 [151]. It is notable that none of these cell lines were in widespread use and that Kuramochi previously had been described as being derived from an undifferentiated neoplasm [151]. Recently the group of Coscia et al. used a proteomic signature to stratify putative HGSOC cell lines into three distinct groups (called groups I-III) [153]. Although the majority of cell lines with a high genetic fidelity to HGSOC were classified as group-I and bore a more epithelial proteome, there were two cell lines recapitulating the genetic features of the disease (59M and TYKNU), which clustered in group-III with a more mesenchymal proteome [153]. On the one hand, there was a striking concordance between the proteomic signature of group-I cell lines and a set of HGSOC patient samples; this signature also clustered closely with cultured fallopian tube epithelial cells. On the other hand, group-III cell lines resembled the signature of immortalized OSE cells. These findings suggest that heterogeneity exists in the proteome of HGSOC, which likely is based on disparate sites of origin (OSE vs. FTSEC) and that this diversity is mirrored in HGSOC cell lines [153].

There is a striking lack of patient-derived cell lines of chemo-resistant HGSOC. The vast majority of cell-lines used to represent platinum-resistant disease have been generated in-vitro by exposing cells to platinum continuously for extended durations of time (usually many months). Although this method often has succeeded in generating cells with many-fold greater resistance to platinum, the type of drug exposure used to elicit the resistance phenotype is unrealistic and fails to resemble what is achievable clinically. As a result, the mechanisms involved in mediating the created resistance likely are different from those acting within the patient’s physiological environment. An ideal study would establish multiple cell lines longitudinally from the same patient so that the molecular and genetic changes that accompany the onset of resistance can be identified. Presently, few such models of HGSOC are known to exist. Set-matched cell lines were established in the UK during the 1980s [154]. These cell lines, known as PEO1/PEO4/PEO6; PEO14/PEO23; PEA1/PEA2, each were established from an individual patient before and after the onset of chemoresistance. PEO14 and PEA1 were established in a chemo-naïve state before the induction of chemotherapy; their counterparts, PEO23 and PEA2, were isolated upon relapse with treatment-resistant disease after 7 and 5 months [154]. PEO1 originated as a first relapse of a patient treated 22 months prior with cisplatin, 5-fluorouracil and chlorambucil [154]. At that stage, the disease was still sensitive to retreatment but upon a second relapse after 10 months, the disease was found to be resistant, with the patient failing to respond following 3 months of an elevated-dose regimen of cisplatin [154]. The second relapse resulted in the generation of PEO4, while PEO6 was generated just 3 months later before the patient succumbed to the disease [154]. Although these cell lines were not assessed in the paper by Domke et al., a subsequent paper by Beaufort et al. assigned them all to be putative HGSOC based on genomic features [155]. This work also established concordance between the morphology of the cell lines in-vitro and their molecular subtype as defined by Tothill et al. [84]. Cells displaying a spindle morphology clustered with the C1 (stromal) tumours, those with rounded morphology with the C5-mesenchymal subtype and the epithelial-like cell lines with the C4 subtype [155].

More recently, a pair of cell lines were developed in Canada from the ascites of the same patient before and after a sequence of cisplatin/topotecan followed by carboplatin/paclitaxel chemotherapy, thus representing relevant clinical acquired resistant to platinum-based chemotherapy. These cells were named OV2295, developed from the patient before receiving therapy and OV2295 (R2), developed about 11 months later upon disease recurrence; OV2295 (R2) were ~16 fold less sensitive to carboplatin [156]. Of interest, these cell lines were developed to growth under 5% oxygen, making it more clinically relevant than the majority of other cell lines, which were developed to growth under air levels of oxygen (~21%) that are non-physiological.

New three-dimensional, organotypic cultures have also been developed that better mimic the in-vivo growth environment compared with growth on plastic [22]. One novel method developed by Kenny et al. involves growing ovarian cancer cells on a multilayered substrate consisting of fibroblasts, mesothelial cells and extracellular matrix [157]. Cells also can be grown in tri-dimensions on a layer of Matrigel^TM^ or using intact peritoneal explants from human or animal sources [158]. Because HGSOC cells have a propensity in-vivo to form spheroids and aggregates that grow in suspension, culture systems can be employed that favor the formation of such structures in-vitro, such as a low-adherence culture surface, spinning flasks or through the use of the hanging-drop method [158].

### 14.2. In-Vivo Models

There are currently few in-vivo model systems for the study of HGSOC, which closely resemble the human disease. Efforts to develop a new generation of accurate murine models have employed contrasting approaches. First, there are the genetically-engineered mouse models (GEMM) that are designed to mirror the same sequence of genetic defects that contribute to human carcinogenesis. Upon induction, these mice begin to develop tumours, either from the ovarian surface epithelium (OSE) or the fallopian tube, that may recapitulate many of the features found in the human disease [22]. This approach allows the disease to be investigated at its earliest stages, something that another methodology cannot replicate [159]. Until recently, a lack of tissue-specific promoters for the putative site of origin for HGSOC hampered the creation of these murine models (recently, the HGSOC site-origin has been mostly traced to the distal fallopian tube) [159]. Historically, models have targeted the OSE using the conditional expression of oncogenes such as KRAS or deletion of tumour suppressors such as TP53, RB, BRCA1/2 and PTEN [22]. This approach, however, has yielded tumours that do not match the histology, marker expression and disease course found in patients [22]. These failures have stimulated the development of a new generation of mouse models with greater emphasis on the fallopian tube as the site of origin for HGSOC carcinogenesis [22]. Recently Kim et al. demonstrated a model using the conditional deletion of DICER and PTEN using the Müllerian specific promotor Amhr2 in loxP-Cre mice [160]. These animals develop high-grade serous adenocarcinoma from the fallopian tube with many histological and molecular similarities to human HGSOC [22,160]. The disease course in these mice also parallels that of the human disease [160]. This model, however, failed to demonstrate emergence from the precursor lesions (STICs) that are thought to represent the earliest stage of HGSOC progression [22]. Another model by Perets and colleagues employs a triple deletion of BRCA, TP53 and PTEN, all mutations found recurrently in patients, under the control of the Pax8 promotor [53]. These transgenic mice developed disease resembling many of the clinical-pathological features of HGSOC from the secretory epithelial cells of the distal fallopian tube (FTSEC) [22,53]. Moreover, the mice presented early onset with precursor lesions in the same locations as the STICs observed in humans [53].

Sherman-Baust et al. also have created a model that uses the expression of the SV40 large T-antigen under the control of the Müllerian-specific Ovgp-1 promotor [161]. In this case, spontaneous neoplastic lesions are seen to originate in the fallopian tube and endometrium [161]. This model furthermore has demonstrated a progression from a precursor lesion resembling the STIC to a highly invasive disease similar to HGSOC [22,161].

Finally, there is the model Zhai and colleagues have generated, involving the deletion of the four tumour suppressor genes BRCA1, TRP53, RB1 and NF1, also using the Ovgp-1 promotor [162]. This model established the essential role of PTEN in the evolution of HGSOC, as animals without PTEN deletion failed to develop tumours even in the presence of mutations to the other three genes [162]. Animals in which all four genes were inactivated presented with STICs that progress to HGSOC or carcinosarcoma, with the presence of widespread metastatic disease in many mice [162]. NF1 deletion was not necessary for this process but the disease arose quicker in animals with all four genes deleted [161]. The existence of these new and improved GEMM offers a promising avenue for addressing many inquiries relating to the early stages of HGSOC pathogenesis as well as providing an opportunity to identify new therapeutic targets and test experimental treatments [22].

Beyond GEMM, there are also in-vivo models that involve the transplantation of human cancer cells into immunodeficient mice (xenografts). Traditionally these have involved the transplantation of cells from human cell lines into athymic (nude) mice, either subcutaneously or orthotopically, such as into the peritoneal cavity or within the ovarian capsule or fallopian tube [22,163]. Because the majority of cell lines previously used to represent HGSOC poorly recapitulate the characteristic features of the disease at the genetic level, a new effort is underway to develop transplantation models using cell lines with high genetic fidelity to HGSOC [151,152]. Unfortunately, many such cell lines grow poorly when transplanted intraperitoneally in nude mice, requiring more severe combined-immunodeficient (SCID) or NOD-SCID IL2R-gamma^null^ (NSG) mice to be used [152,164], something that is not considered desirable in view of the important contribution of immune cells to the HGSOC micro-environment.

An alternative approach that has been highlighted recently involves the transplantation of minced tumour fragments freshly isolated from human patients into orthotopic sites in immunodeficient mice [159]. Many studies have evaluated the efficacy and suitability of this method, also known as patient-derived xenografts (PDXs) and have generally described a high degree of resemblance to the disease observed within the original patient, including the preservation of intra-tumour phenotypic heterogeneity [22,165]. The efficiency of transplantation has been seen to depend upon the degree of immunodeficiency of the recipient mouse and the site of injection [22]. One study has shown that tumours that engraft more readily are associated with a poorer prognosis for the patient from whom they were derived [166]. These PDXs might be used clinically to test novel therapies and to predict the response of the patient to treatment [22]. Nonetheless, some deficiencies have been underlined, regarding these models. For one, they represent only the late stage of the disease due to the injection of the cells or tissue directly within the peritoneal cavity [159]. On the other hand, variation exists between the models using SCID versus NSG mice. In the SCID mice, mouse stroma rapidly replaced the human stroma, whereas in the case of NSG mice, the human stroma remained in the long-term [159].

## 15. Conclusions

In spite of its uncommon incidence, ovarian cancer represents a salient public health concern because of its dismal long-term survival outcomes, which have not improved substantively in decades. Ovarian cancer is conceived more appropriately as a spectrum of malignancy differing in terms of histology, clinical behaviour and molecular-genetic features. Of the numerous subtypes, HGSOC is the most common and by far the deadliest. With few early warning signs and an unspecific symptomology, HGSOC rarely is diagnosed in its early stages. The majority of patients thus will present with a disease that already has disseminated widely within the peritoneal cavity, significantly complicating the task of surgical resection. Moreover, while the initial response to the frontline platinum-based chemotherapy is typically excellent, recurrence is almost assured, with a disease that eventually will attain resistance to treatment. Recently, PARP inhibitors and antiangiogenic agents entered clinical use after demonstrating efficacy in enhancing progression-free survival in the latest trials. Although long postulated to be of ovarian origin, the majority of HGSOC cases are now thought to be derived from the secretory epithelial cells of the distal fallopian tube. Genetically, there are few recurrent driver mutations in HGSOC other than those involving P53 and a much more prominent role for genomic instability and gene copy number alterations. HGSOC contains its own spectrum of molecular diversity, with a range of subtypes being identified on the basis of contrasting patterns of gene expression. Ultimately, while in many areas HGSOC continues to elude our understanding, the recent pace of discovery portends well for the fate of individuals likely to be diagnosed with this disease in the future.

## Abbreviations

ARID1A	AT-Rich Interaction Domain 1A
ATM	ATM Serine/Threonine Kinase
AKT	AKT Serine/Threonine Kinase
ATR	ATR serine/threonine kinase
AUC	Area Under the Curve
BARD1	BRCA1 Associated RING Domain 1
BECN1	Beclin 1
BER	Base Excision Repair
BRAF	B-Raf Proto-Oncogene, Serine/Threonine Kinase
BRCA	BRCA, DNA Repair Associated
BRIP1	BRCA1 Interacting Protein C-Terminal Helicase 1
CA125	Cancer Antigen 125
CCNE1	Cyclin E1
CDKN2A	Cyclin Dependent Kinase Inhibitor 2A
CDKN2D	Cyclin Dependent Kinase Inhibitor 2D
CDK12	Cyclin Dependent Kinase 12
CHEK2	Checkpoint Kinase 2
CICs	Cortical Inclusion Cysts
CK7	Cytokeratin 7
CK20	Cytokeratin 20
CNAs	Copy Number Alterations
CT	Computer Tomography
CTNNB1	Catenin Beta 1
DICER	Dicer 1, Ribonuclease III
DNA	Deoxyribonucleic acid
EMA	European Medicines Agency
EOC	Epithelial Ovarian Cancer
ER	Estrogen Receptor
FDA	Food and Drug Administration
FIGO	International Federation of Gynecology and Obstetrics
FOXM1	Forkhead Box M1
FTSEC	Secretory Epithelial Cells of the Distal Fallopian Tube
GAB2	GRB2 Associated Binding Protein 2
GABRA6	Gamma-Aminobutyric Acid Type A Receptor Alpha6 Subunit
g-H2AX	Gamma H2A histone family member X
GEMM	Genetically Engineered Mouse Models
GOG	Gynecologic Oncology Group
GWAS	Genome Wide Association Studies
HDM2	HDM2 Proto-Oncogene
HGSOC	High Grade Serous Ovarian Cancer
HIF-1a	Hypoxia Inducible Factor 1 Subunit Alpha
HMGA2	High Mobility Group AT-Hook 2
HNF1b	HNF1 Homeobox B
HOXA9	Homeobox A9
IARC	International Agency for Research on Cancer
KRAS	KRAS Proto-Oncogene, GTPase
LC3	Microtubule Associated Protein 1 Light Chain 3 Alpha
LOH	Loss of Heterozygosity
MAPK	Mitogen-Activated Protein Kinase 1
MECOM	MDS1 And EVI1 Complex Locus
MRI	Magnetic Resonance Imaging
MRE11A	MRE11 Homolog, Double Strand Break Repair Nuclease
MYC	MYC Proto-Oncogene, BHLH Transcription Factor
NACT	Neoadjuvant Chemotherapy
NCI	American National Cancer Institute
NF1	Neurofibromin 1
NSG	NOD-SCID IL2R-gamma^null^
OSE	Ovarian Surface Epithelium
PALB2	Partner and Localizer of BRCA2
PARP	Poly (ADP-Ribose) Polymerase
PAX8	Paired Box 8
PDXs	Patient Derived Xerographs
PET	Positron-Emission Tomography
PI3K	Phosphatidylinositol-4,5-Bisphosphate 3-Kinase Catalytic Subunit
PIK3CB	Phosphatidylinositol-4,5-Bisphosphate 3-Kinase Catalytic Subunit Beta
PIK3CA	Phosphatidylinositol-4,5-Bisphosphate 3-Kinase Catalytic Subunit Alpha
PR	Progesterone Receptor
PTEN	Phosphatase and Tensin Homolog
P53	Tumor Protein P53
RAD50	RAD50 Double Strand Break Repair Protein
RAD51C	RAD51 Paralog C
RAS	KRAS Proto-Oncogene, GTPase
RB	RB Transcriptional Corepressor
ROS	Radical Oxygen Species
RTK	Receptor Tyrosine Kinases
SCID	Severe Combined Immunodeficient
SEER	Surveillance Epidemiology and End Results
SET	Solid pseudo-Endometrioid and/or Transitional Cell Carcinoma-like
SNPs	Single Nucleotide Polymorphisms
STIC	Serous Tubal Intra Epithelial Carcinoma
TCGA	The Tumor Genome Atlas
UK	United Kingdom
VEGF-A	Vascular Endothelial Growth Factor A
WDFY2	WD Repeat and FYVE Domain Containing 2
WHO	World Health Organization
WNT	Wnt Family Member
WT1	Wilms Tumor 1
